# Supplementary material for: TCF-1 Is Required for CD4 T Cell Persistence Functions during AlloImmunity
Source: Int J Mol Sci. 2023 Feb 21;24(5):4326. doi: 10.3390/ijms24054326 (PMC10002223; doi:10.3390/ijms24054326)
Supplement: Supplementary file 1 [file ijms-24-04326-s001.zip › ijms-2080648-supplementary.pdf]

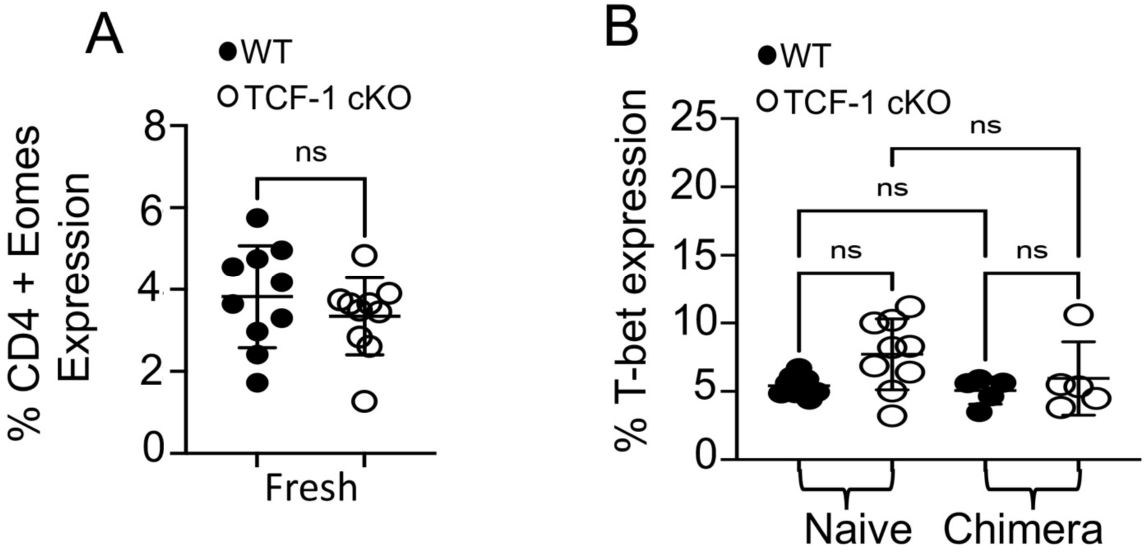

Figure S1 (related to Figure 1) Loss of TCF-1 does not alter Eomes expression in CD4 T cells but upregulates T-bet expression in a cell-extrinsic manner: (**A**) Naïve WT or TCF-1 cKO donor mice were euthanized, splenocytes were obtained and stained for flow cytometry, and run on a BD LSRFortessa flow cytometer. Statistical analysis of the percentage of CD4 T cells expressing Eomes. (**B**) Thy1.1 mice were lethally irradiated and reconstituted with a 1:4 (WT:TCF-1 cKO) mixture of bone marrow cells. At 9 weeks, blood was checked by flow cytometry to ensure reconstitution, and at 10 weeks, flow cytometry phenotyping was performed. WT donor cells were identified by CD45.1, while TCF-1 cKO donor cells were identified by CD45.2. Percent of chimeric and naïve CD4 T cells expressing T-bet is shown. All data are shown as individual points with mean and SD, all data were analyzed with Student's t-test, or one-way ANOVA (depending on the data groups). For naïve mice, N=3-5 per group of mice and combined data from 2-3 experiments are shown. For chimeric mice, N=5 per group of mice and one experiment is shown (carried out once). * Means p-value ≤ 0.05, ** means p-value ≤ 0.01, and *** means p-value ≤ 0.001, **** means p-value≤ 0.0001.

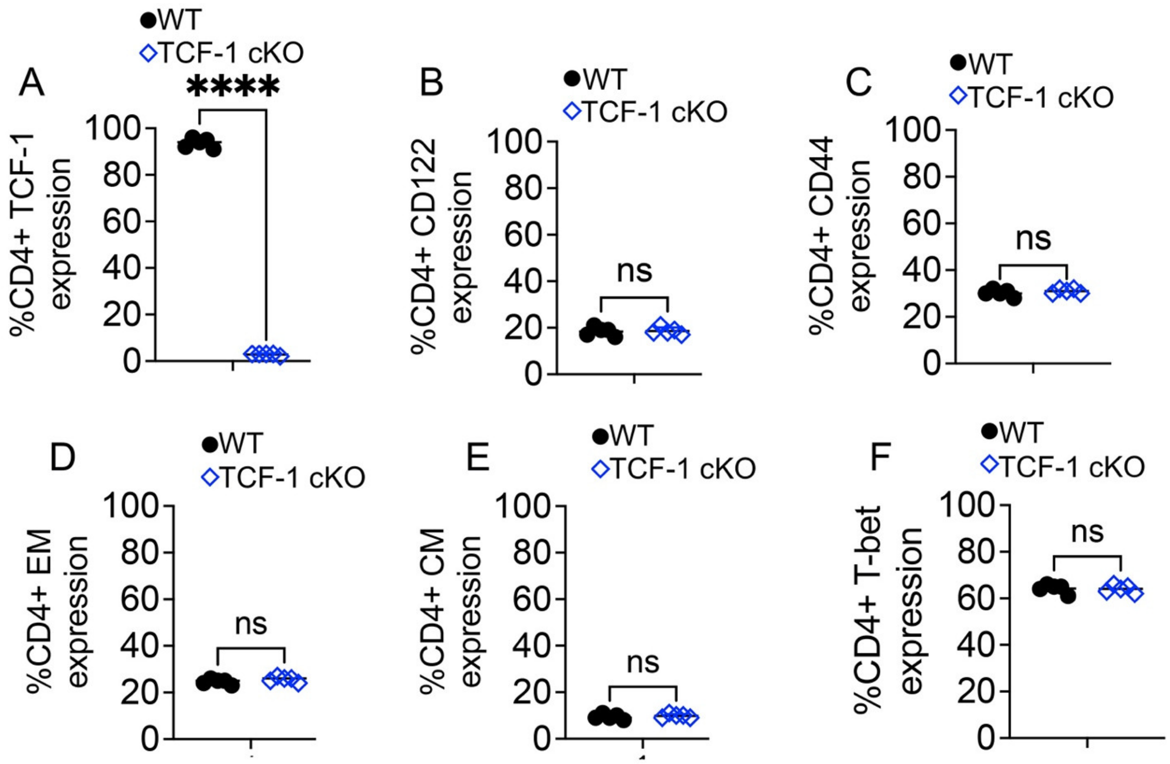

Figure S2 (related to Figure 1) TCF-1 regulates CD4 T cell activation markers during mixed chimera bone marrow transplantation in a cell-extrinsic manner: Thy1.1 mice were lethally irradiated and reconstituted with a 1:4 (WT:TCF-1 cKO) mixture of bone marrow cells or control (WT:CD4re+/+) mixture of bone marrow cells. At 9 weeks, blood was checked by flow cytometry to ensure reconstitution, and at 10 weeks, flow cytometry phenotyping was performed. WT donor cells were identified by CD45.1 as WT and CD45.2 as TCF-1 cKO mice (**A**) Percentage of mixed bone marrow chimeric cells from derived CD4 T cells from (WT:TCF-1cKO) CD4 T cells expressing TCF-1. (**B**) Percentage of mixed chimera derived CD4 T cells from (WT:TCF-1 cKO) expressing CD122. (**C**) Percentage of mixed chimera derived CD4 T cells from (WT:TCF-1 cKO) expressing CD44. (**D**) Percentage of mixed chimera derived CD4 T cells from (WT:TCF-1 cKO) expressing the effector memory phenotype. (**E**) Percentage of mixed chimera derived CD4 T cells from (WT:TCF-1 cKO) expressing the central memory phenotype. (**F**) Percentage of mixed chimera derived CD4 T cells from (WT:TCF-1 cKO) expressing the central memory phenotype. One of three experiments presented.

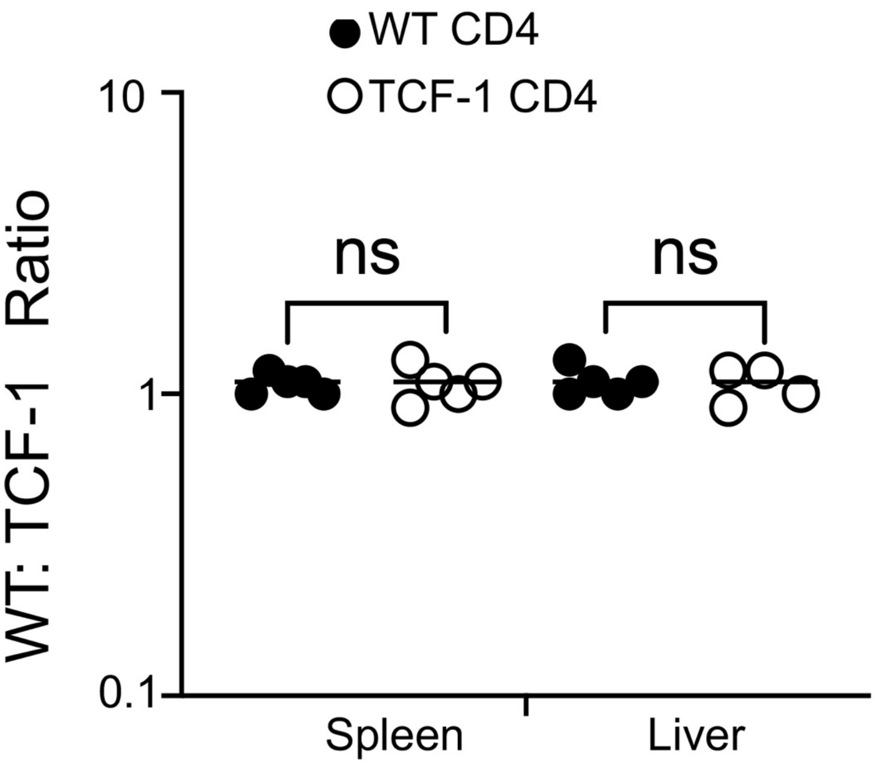

Figure S3 (related to Figure 3) The lack of TCF-1 on CD4 T cells has no impact on donor T cell migration to GvHD target organs: Irradiated BALB/c mice were allo-HSCT-transplanted and injected with FACS-sorted WT or TCF-1 cKO $CD4^+$ T cells mixed at a 1:1 ratio. FACS analysis of sorted T cells pre-transplant is shown. At day 7 post-BMT, the spleen and liver were examined for donor CD4 T cells from either WT or TCF-1 cKO mice. One of two experiments is shown.

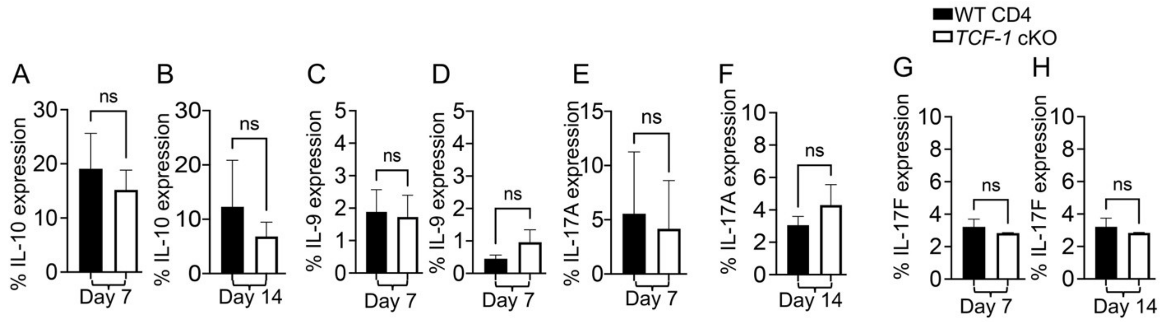

Figure S4 (related to Figure 7) TCF-1 does not regulate all pro inflammatory cytokines during alloimmunity: Recipient BALB/c mice were allotransplanted with WT or TCF-1 cKO donor CD4 T cells, as before. Serum was obtained from cardiac blood of euthanized recipient mice at day 7 and day 14 post-transplant, and was tested using a LEGENDplex multiplex ELISA kit. Serum concentration (pg/mL) over time for WT versus TCF-1 cKO-transplanted mice. (**A-B**) Day 7 and at day 14 post-transplant IL-10. (**C-D**) Day 7 and day 14 IL-9, IFN-γ. (**E-F**) Day 7 and day 14 IL-17A. (**G-H**) Day 7 and day 14 IL-17F. All data were analyzed with Student's t-test. * Means p-value ≤ 0.05, ** means p-value ≤ 0.01, and *** means p-value ≤ 0.001, **** means p-value≤ 0.0001.

Apoptotic processes and Cell death related pathways. Pre-transplant

Source	Term name	Term ID	FDR	-Log10 FDR	Intersection Size	Intersections
GO:BP	**Cell death**	GO:0008219	4.34E-13	12.36216201	118	4930453N24RIK,ACER2,ACSL5,ADAM10,AIFM1,AK6,AP2B1,API5,ASAH1,ATG3,BIRC3,BTK,CACYBP,CAMK2D,CASP1,CASP8,CAT,CBL,CD24A,CD274,CD38,CD3E,CD5,CDC42,CFDP1,CLIP3,CRKL,CTNNB1,CTSC,DAPL1,DDX20,DDX5,DNAJA1,EIF2S1,ELMO1,ETS1,FSTL1,GM16513,GNAI3,GZMA,HIF1A,HMGB2,HSH2D,HSP90AA1,HSP90B1,HSPA5,HSPA9,IFIT2,IFIT3,IKBKE,IPMK,ITGB1,IVNS1ABP,JTB,KIT,KLK8,KLRK1,KPNA1,LCK,LYN,MAD2L1,MALT1,MCL1,MDM2,MFF,MRE11A,MSH6,NCK1,NCL,NDUFA13,NDUFS1,NFKB1,NOX4,NPM1,NSMAF,NSMF,NTRK3,PDCD4,PDIA3,PHIP,PIK3CG,PLSCR1,PPP2R5C,PRDM11,PRKCB,PTEN,PTGES3,PTPRC,QRICH1,RASSF5,RBM5,REST,RSL1D1,SERINC3,SERPINB9,SET,SH3KBP1,SLC40A1,SNX6,SORL1,SP100,SPATA2,SRGN,TAF10,TARDBP,TAX1BP1,TMEM123,TNFAIP8,TNFAIP8L1,TOMM70A,TOPORS,TRP53INP1,TXNRD1,VPS35,VPS4B,WRN,YWHAZ,ZFAND6
GO:BP	**Programmed cell death**	GO:0012501	1.03E-11	10.98665277	108	4930453N24RIK,ACER2,ACSL5,ADAM10,AIFM1,AK6,API5,ASAH1,ATG3,BIRC3,BTK,CAMK2D,CASP1,CASP8,CAT,CBL,CD24A,CD274,CD38,CD3E,CD5,CDC42,CFDP1,CLIP3,CRKL,CTNNB1,CTSC,DAPL1,DDX20,DDX5,DNAJA1,EIF2S1,ELMO1,ETS1,FSTL1,GM16513,GNAI3,GZMA,HIF1A,HMGB2,HSH2D,HSP90AA1,HSP90B1,HSPA5,IFIT2,IFIT3,IKBKE,IPMK,ITGB1,IVNS1ABP,JTB,KIT,KLRK1,KPNA1,LCK,LYN,MAD2L1,MALT1,MCL1,MDM2,MFF,MRE11A,MSH6,NCK1,NCL,NDUFA13,NDUFS1,NFKB1,NOX4,NPM1,NSMAF,NSMF,NTRK3,PDCD4,PDIA3,PHIP,PIK3CG,PLSCR1,PPP2R5C,PRDM11,PRKCB,PTEN,PTPRC,QRICH1,RASSF5,RBM5,REST,RSL1D1,SERINC3,SERPINB9,SET,SH3KBP1,SLC40A1,SNX6,SP100,SPATA2,SRGN,TAF10,TARDBP,TAX1BP1,TNFAIP8,TNFAIP8L1,TOMM70A,TOPORS,TRP53INP1,VPS35,WRN,ZFAND6
GO:BP	**Apoptotic process**	GO:0006915	4.79E-11	10.32000927	104	4930453N24RIK,ACER2,ACSL5,ADAM10,AIFM1,AK6,API5,ATG3,BIRC3,BTK,CAMK2D,CASP1,CASP8,CAT,CBL,CD24A,CD274,CD38,CD3E,CD5,CDC42,CFDP1,CLIP3,CRKL,CTNNB1,CTSC,DAPL1,DDX20,DDX5,DNAJA1,EIF2S1,ELMO1,ETS1,FSTL1,GM16513,GNAI3,GZMA,HIF1A,HMGB2,HSH2D,HSP90AA1,HSP90B1,HSPA5,IFIT2,IFIT3,IKBKE,ITGB1,IVNS1ABP,JTB,KLRK1,KPNA1,LCK,LYN,MAD2L1,MALT1,MCL1,MDM2,MFF,MRE11A,MSH6,NCK1,NCL,NDUFA13,NDUFS1,NFKB1,NOX4,NPM1,NSMAF,NSMF,NTRK3,PDCD4,PDIA3,PHIP,PIK3CG,PLSCR1,PPP2R5C,PRDM11,PRKCB,PTEN,PTPRC,QRICH1,RASSF5,RBM5,REST,RSL1D1,SERINC3,SERPINB9,SET,SH3KBP1,SLC40A1,SNX6,SP100,SRGN,TAF10,TARDBP,TAX1BP1,TNFAIP8,TNFAIP8L1,TOMM70A,TOPORS,TRP53INP1,VPS35,WRN,ZFAND6
GO:BP	**Apoptotic signaling pathway**	GO:0097190	0.000288143	3.540392486	34	ACSL5,AIFM1,CASP1,CASP8,CD24A,CD3E,CD5,CTNNB1,CTSC,DAPL1,DDX5,DNAJA1,GNAI3,HIF1A,HMGB2,IKBKE,IVNS1ABP,LCK,MCL1,MDM2,MFF,MSH6,NCK1,NDUFA13,PDIA3,PHIP,PLSCR1,PPP2R5C,PTEN,PTPRC,QRICH1,SERINC3,SP100,TOPORS
GO:BP	**Lymphocyte apoptotic process**	GO:0070227	0.006491763	2.187637315	9	CASP8,CD24A,CD274,CRKL,HIF1A,HSH2D,LYN,PTEN,SERPINB9
GO:BP	**Programmed necrotic cell death**	GO:0097300	0.00918796	2.036780913	6	ASAH1,BIRC3,CASP1,CASP8,IPMK,SPATA2
GO:BP	**Intrinsic apoptotic signaling pathway**	GO:0097193	0.015995515	1.79600176	17	AIFM1,DDX5,DNAJA1,HIF1A,IKBKE,IVNS1ABP,LCK,MCL1,MDM2,MSH6,NCK1,NDUFA13,PLSCR1,PPP2R5C,QRICH1,SERINC3,TOPORS
GO:BP	**Mitochondrial fragmentation involved in apoptotic process**	GO:0043653	0.022839267	1.641317837	3	ATG3,MFF,VPS35

T cell mediated processes related pathways. Pre-transplant

Source	Term Name	Term ID	FDR	-Log10 FDR	Intersection size	Intersections
GO:BP	**T cell activation**	GO:0042110	8.10E-07	6.091298982	37	AP3B1,CD24A,CD274,CD3D,CD3E,CD47,CD48,CD5,CD55,CD6,CD86,CD8A,CEACAM1,CTNNB1,DOCK2,FGL2,HSH2D,HSP90AA1,IL4I1,IRF4,KIT,LAT,LCK,LCP1,LFNG,MALT1,NCK1,PTPN22,PTPRC,SIT1,SLA2,SP3,TNFRSF13C,VCAM1,ZAP70,ZBTB1,ZP3
GO:BP	**T cell mediated immunity**	GO:0002456	1.96E-06	5.707943223	15	CD24A,CD55,CD8A,CEACAM1,CR1L,CTSC,HPRT,IL4I1,KDM5D,MALT1,PTPRC,SERPINB9,STX7,ZBTB1,ZP3
GO:BP	**T cell proliferation**	GO:0042098	0.000137258	3.862462509	18	CD24A,CD274,CD3E,CD55,CD6,CD86,CEACAM1,CTNNB1,DOCK2,IL4I1,MALT1,NCK1,PTPN22,PTPRC,TNFRSF13C,VCAM1,ZAP70,ZP3
GO:BP	**T cell receptor signaling pathway**	GO:0050852	0.001551673	2.809199679	12	CD3E,CEACAM1,CRKL,ELF1,KCNN4,LCK,MALT1,PDE4B,PTPN22,PTPRC,SLA2,ZAP70
GO:BP	**T cell differentiation**	GO:0030217	0.001722456	2.763851756	19	AP3B1,CD3D,CD3E,CD8A,CTNNB1,DOCK2,FGL2,HSP90AA1,IL4I1,IRF4,KIT,LCK,LFNG,MALT1,PTPN22,PTPRC,SP3,ZAP70,ZBTB1
GO:BP	**I-kappaB kinase/NF-kappaB signaling**	GO:0007249	0.014041503	1.85258641	15	BTK,CASP1,CASP8,CTNNB1,DDX1,DDX21,IKBKE,MALT1,PRKCB,TANK,TBK1,TRIM12A,TRIM30A,TRIM30D,ZFAND6
KEGG	**T cell receptor signaling pathway**	KEGG:04660	0.008130453	2.08988527	14	CD3D,CD3E,CD8A,CDC42,LAT,LCK,MALT1,NCK1,NFATC3,NFKB1,NRAS,PTPRC,TEC,ZAP70
KEGG	**Th17 cell differentiation**	KEGG:04659	0.035251567	1.452821575	12	AHR,CD3D,CD3E,HIF1A,HSP90AA1,IRF4,JAK1,LAT,LCK,NFATC3,NFKB1,ZAP70

Cytokine response and production related genes. Pre-transplant

Source	Term name	Term ID	FDR	-Log10 FDR	Intersection size	Intersections
GO:BP	**Response to cytokine**	GO:0034097	6.55E-08	7.183898653	65	9930111J21RIK1,ACTR2,ACTR3,ADAM10,AK6,ASAH1,B3GNT2,BTK,CACYBP,CASP1,CASP8,CCR3,CD24A,CD274,CD38,CD47,CDC42,CEACAM1,CRKL,CTNNB1,CXCR6,F830016B08RIK,GBP3,GBP7,GBP9,GPS2,HIF1A,HNRNPU,HSPA5,IFIT2,IFIT3,IKBKE,JAK1,KIF5B,KIT,LSM14A,MCL1,MRC1,MTF2,MYNN,NCL,NDUFA13,NFKB1,NFYB,OASL2,PDIA3,PLSCR1,PTPRC,PYDC3,PYHIN1,RPS16,SAMHD1,SETD2,SP100,SPATA2,SRSF3,STAT4,SYNCRIP,TANK,TBK1,TNFRSF13C,VCAM1,YTHDF2,YTHDF3,ZFAND6
GO:BP	**Regulation of cytokine production**	GO:0001817	3.76E-07	6.42525599	47	ATP2B1,BANK1,BTK,CASP1,CASP8,CD24A,CD274,CD2AP,CD3E,CD47,CD55,CD6,CEACAM1,CHIL3,CYBB,DDX1,DDX21,ELF1,HDAC2,HIF1A,HMGB2,HSP90AA1,IQGAP1,IRF4,KIT,KLRK1,LTBP1,LYN,MALT1,MCOLN2,NCL,NFKB1,PDCD4,PDE4B,POLR3B,PTPN22,PTPRC,SERPINB1A,SETD2,SORL1,SPTBN1,SRGN,TBK1,TNFRSF13C,TOMM70A,TRIM30A,ZP3
GO:BP	**Regulation of cytokine-mediated signaling pathway**	GO:0001959	0.001456837	2.836589162	12	CASP1,CD24A,GPS2,HIF1A,IKBKE,LSM14A,PTPRC,SAMHD1,SPATA2,TBK1,YTHDF2,YTHDF3
GO:BP	**Positive regulation of interleukin-2 production**	GO:0032743	0.003289949	2.482810855	6	CD3E,IRF4,MALT1,PDE4B,PTPRC,SPTBN1
GO:BP	**Regulation of type I interferon-mediated signaling pathway**	GO:0060338	0.004253895	2.371213195	6	IKBKE,LSM14A,SAMHD1,TBK1,YTHDF2,YTHDF3
WP	**IL-5 signaling pathway**	WP:WP151	0.032475753	1.488440773	11	BTK,CBL,CRKL,CTNNB1,JAK1,LYN,NFKB1,PIK3CG,PRKCB,SDCBP,YWHAZ

Figure S5 (related to Figure 8). Loss of TCF-1 alters gene expression of pre-transplant donor CD4 T cells: Gene ontology enrichment analysis of pre-transplant CD4 T cell samples was conducted using the g: Profiler toolset; g:GOSt tool. Tables showing the cell death and apoptotic process-related pathways and the involved genes that were altered due to loss of TCF-1, the T cell-mediated process-related pathways and the involved genes that were altered due to loss of TCF-1, and the cytokine response and production-related pathways and the involved genes that were altered due to loss of TCF-1 are provided.

Apoptotic processes and cell death related pathways. Post-transplant

Source	Term name	Term ID	FDR	-Log10 FDR	Intersection size	Intersections
GO:BP	**Cell death**	GO:0008219	5.45E-07	6.263634994	101	ADAMTSL4,ALPK2,ANK2,ANXA6,APBB2,ARHGEF1,ARRB1,ATP2A3,BTG2,CARD11,CARD6,CCL3,CCL5,CCR5,CD27,CD28,CD34,CD3E,CD3G,CD5,CHD8,CORO1A,CTLA4,CXCR2,CXCR3,CYFIP2,DDIT4,DYNLT1B,EEF1A1,EGR1,ERBB4,ETS1,FN1,FSTL1,GIMAP5,GNAQ,HGF,HMGB1,ID3,IGF1,IKBKG,IKZF3,IL27RA,IL2RB,IPMK,ITGA6,ITM2C,ITSN1,LAPTM5,LCK,LSP1,MDM4,MICAL1,MPV17L,NDNF,NFKB1,NOA1,NOX4,NUPR1,PAK2,PAX6,PDCD1,PDGFRB,PGLYRP1,PINK1,PLA2R1,PLSCR2,PRDM11,PRKCH,PRKCQ,PRKD2,PRLR,RASSF5,RHOT2,RPS3,RPS3A1,SGK3,SGPL1,SHISA5,SIX4,SLC7A11,SLIT2,TBC1D24,TCIRG1,TEAD2,TGM2,TMEM214,TNFAIP3,TNFAIP8,TNFAIP8L2,TNFRSF10B,TNFRSF1B,TRAF1,TSC22D3,TXNIP,UBB,UCP2,USP17LA,WNT4,YAP1,YPEL3
GO:BP	**Programmed cell death**	GO:0012501	1.97075E-06	5.705368497	93	ADAMTSL4,ALPK2,ANK2,ANXA6,APBB2,ARRB1,ATP2A3,BTG2,CARD11,CARD6,CCL3,CCL5,CCR5,CD27,CD28,CD3E,CD3G,CD5,CHD8,CORO1A,CTLA4,CXCR2,CXCR3,CYFIP2,DDIT4,EGR1,ERBB4,ETS1,FN1,FSTL1,GIMAP5,GNAQ,HGF,HMGB1,ID3,IGF1,IKBKG,IKZF3,IL27RA,IL2RB,IPMK,ITGA6,ITM2C,ITSN1,LAPTM5,LCK,LSP1,MDM4,MICAL1,MPV17L,NDNF,NFKB1,NOA1,NOX4,NUPR1,PAK2,PDCD1,PDGFRB,PGLYRP1,PINK1,PLA2R1,PLSCR2,PRDM11,PRKCH,PRKCQ,PRLR,RASSF5,RHOT2,RPS3,RPS3A1,SGK3,SGPL1,SHISA5,SIX4,SLC7A11,SLIT2,TCIRG1,TGM2,TMEM214,TNFAIP3,TNFAIP8,TNFAIP8L2,TNFRSF10B,TNFRSF1B,TRAF1,TSC22D3,TXNIP,UBB,UCP2,USP17LA,WNT4,YAP1,YPEL3
GO:BP	**Apoptotic process**	GO:0006915	1.97075E-06	5.705368497	91	ADAMTSL4,ALPK2,ANK2,ANXA6,APBB2,ARRB1,ATP2A3,BTG2,CARD11,CARD6,CCL3,CCL5,CCR5,CD27,CD28,CD3E,CD3G,CD5,CHD8,CORO1A,CTLA4,CXCR2,CXCR3,CYFIP2,DDIT4,EGR1,ERBB4,ETS1,FN1,FSTL1,GIMAP5,GNAQ,HGF,HMGB1,ID3,IGF1,IKBKG,IKZF3,IL27RA,IL2RB,ITGA6,ITM2C,ITSN1,LCK,LSP1,MDM4,MICAL1,MPV17L,NDNF,NFKB1,NOA1,NOX4,NUPR1,PAK2,PDCD1,PDGFRB,PGLYRP1,PINK1,PLA2R1,PLSCR2,PRDM11,PRKCH,PRKCQ,PRLR,RASSF5,RHOT2,RPS3,RPS3A1,SGK3,SGPL1,SHISA5,SIX4,SLC7A11,SLIT2,TCIRG1,TGM2,TMEM214,TNFAIP3,TNFAIP8,TNFAIP8L2,TNFRSF10B,TNFRSF1B,TRAF1,TSC22D3,TXNIP,UBB,UCP2,USP17LA,WNT4,YAP1,YPEL3
GO:BP	**Apoptotic signaling pathway**	GO:0097190	0.00784763	2.105261507	30	ANXA6,ATP2A3,CD27,CD28,CD3E,CD5,DDIT4,HGF,IGF1,IKBKG,ITGA6,ITM2C,LCK,MDM4,MPV17L,NUPR1,PAK2,PINK1,RHOT2,RPS3,SGK3,SGPL1,SHISA5,TNFAIP3,TNFRSF10B,TNFRSF1B,TRAF1,UBB,WNT4,YAP1
GO:BP	**Regulation of T cell apoptotic process**	GO:0070232	0.020593151	1.686277194	5	CCL5,CD27,PDCD1,PRKCQ,TSC22D3

T cell mediated processes related pathways. Post-transplant

Source	Term Name	Term ID	FDR	-Log10 FDR	Intersection size	Intersections
GO:BP	**T cell activation**	GO:0042110	4.29E-13	12.36748036	50	BATF,CARD11,CCL5,CD2,CD244,CD27,CD28,CD3D,CD3E,CD3G,CD4,CD5,CORO1A,CTLA2A,CTLA4,DLG1,EGR1,FKBP1A,GIMAP5,GM13285,GPR183,HMGB1,ICOS,IGF1,IL27RA,IL2RG,ITGAL,JAK3,LAG3,LAPTM5,LAX1,LCK,PREX1,PRKCQ,PSMB11,PTPN6,RAC2,RPS3,SASH3,SLAMF6,TCIRG1,TESPA1,TIGIT,TNFAIP8L2,TNFRSF1B,TOX,TXK,WNT4,ZBTB32,ZFP36L2
GO:BP	**T cell proliferation**	GO:0042098	8.05E-08	7.094021133	24	CARD11,CCL5,CD244,CD28,CD3E,CD4,CORO1A,CTLA4,DLG1,FKBP1A,HMGB1,IGF1,IL27RA,ITGAL,JAK3,LAPTM5,PRKCQ,PTPN6,RAC2,RPS3,SASH3,TNFRSF1B,WNT4,ZBTB32
GO:BP	**T cell differentiation**	GO:0030217	3.64E-07	6.438323061	27	BATF,CARD11,CD27,CD28,CD3D,CD3E,CD3G,CD4,CTLA2A,CTLA4,EGR1,GIMAP5,GPR183,HMGB1,IL2RG,LAG3,LCK,PREX1,PSMB11,SASH3,SLAMF6,TCIRG1,TESPA1,TOX,TXK,WNT4,ZFP36L2
GO:BP	**T cell receptor signaling pathway**	GO:0050852	7.9483E-06	5.099723463	16	BTNL9,CARD11,CD247,CD28,CD3E,IKBKG,LAPTM5,LCK,PDE4D,PRKD2,PTPN6,RPS3,SKINT8,TESPA1,TXK,UBASH3A
KEGG	**T cell receptor signaling pathway**	KEGG:04660	3.6079E-06	5.442750078	18	CARD11,CD247,CD28,CD3D,CD3E,CD3G,CD4,CTLA4,DLG1,ICOS,IKBKG,LCK,NFATC1,NFKB1,PAK2,PDCD1,PRKCQ,PTPN6
KEGG	**Th1 and Th2 cell differentiation**	KEGG:04658	0.00062576	3.203595176	13	CD247,CD3D,CD3E,CD3G,CD4,IKBKG,IL2RB,IL2RG,JAK3,LCK,NFATC1,NFKB1,PRKCQ
KEGG	**Th17 cell differentiation**	KEGG:04659	0.00087648	3.057258416	14	CD247,CD3D,CD3E,CD3G,CD4,IKBKG,IL27RA,IL2RB,IL2RG,JAK3,LCK,NFATC1,NFKB1,PRKCQ

Cytokine and chemokine related pathways. Post transplant

Source	Term Name	Term ID	FDR	-Log10 FDR	Intersection Size	Intersections
GO:BP	**Cytokine production**	GO:0001816	5.21E-13	12.28293966	61	ACP5,ADCY7,ALOX5,APPL1,ARID5A,ARRB1,BATF,BTNL9,CARD11,CCL3,CCL4,CCL5,CCR5,CD2,CD200,CD244,CD27,CD28,CD34,CD3E,DDX58,EGR1,EPHB2,EPX,FERMT1,FLOT1,FN1,FURIN,GBP5,GIMAP5,HGF,HMGB1,IGF1,IL16,IL27RA,LAG3,LAPTM5,LPL,LTB,LTBP1,MMP12,NFKB1,PDE4D,PGLYRP1,PLA2R1,POLA1,PRKCQ,PRKD2,PTPN6,RPS3,SASH3,SKINT8,SLAMF6,SYT11,TICAM2,TIGIT,TNFAIP3,TNFRSF1B,TXK,UBASH3A,ZBTB32
GO:BP	**Response to cytokine**	GO:0034097	8.35E-08	7.078314145	66	ABCD4,ACP5,AKAP6,APPL1,BSPRY,CCL3,CCL4,CCL5,CCR5,CD300LF,CD4,CDC42EP2,CORO1A,CSF2RB,CSF2RB2,CXCR2,CXCR3,CXCR5,DDX58,EGR1,ENAH,F830016B08RIK,FKBP1A,FN1,GBP5,GM13285,GM4841,GSTT2,H2-Q7,HMGB1,HYAL3,IFITM1,IFITM2,IL27RA,IL2RB,IL2RG,JAK3,LAPTM5,LDLRAP1,LSP1,MMP12,MYLK3,NFAT5,NFKB1,PAX6,PID1,PRLR,PRPF8,PTPN6,PTPRT,PYDC3,RPL3,RPLP0,RPS2,RPS3,SLIT2,SMARCA5,TCIRG1,TICAM2,TNFRSF1B,TRAF1,TRBV13-2,TXK,UGCG,YAP1,ZFP36L2
GO:BP	**Cytokine-mediated signaling pathway**	GO:0019221	1.97075E-06	5.705368497	33	APPL1,CCL3,CCL4,CCL5,CCR5,CD300LF,CD4,CSF2RB,CSF2RB2,CXCR2,CXCR3,CXCR5,EGR1,FKBP1A,GM13285,IFITM1,IFITM2,IL27RA,IL2RB,IL2RG,JAK3,LAPTM5,MMP12,PRLR,PTPN6,SLIT2,TICAM2,TNFRSF1B,TRAF1,TRBV13-2,TXK,UGCG,YAP1
GO:BP	**Interleukin-2 production**	GO:0032623	3.7298E-06	5.428314308	12	CARD11,CD28,CD34,CD3E,LAG3,LAPTM5,PDE4D,PRKCQ,PRKD2,RPS3,SASH3,TNFAIP3
GO:BP	**Interferon-gamma production**	GO:0032609	1.26119E-05	4.899219667	15	ARID5A,CD2,CD244,CD27,CD3E,GIMAP5,HMGB1,IL27RA,LAPTM5,PDE4D,PGLYRP1,SASH3,SLAMF6,TICAM2,TXK
GO:BP	**Regulation of tumor necrosis factor production**	GO:0032680	9.37754E-05	4.027910849	17	ACP5,ARID5A,CCL3,CCL4,CCR5,CD2,CD34,DDX58,EPHB2,HMGB1,IGF1,IL27RA,LPL,PTPN6,SASH3,SYT11,TNFAIP3
GO:BP	**Regulation of interleukin-6 production**	GO:0032675	0.0012477	2.903889763	14	ARID5A,ARRB1,CCR5,DDX58,HGF,HMGB1,IL16,IL27RA,LAPTM5,LPL,PTPN6,SYT11,TICAM2,TNFAIP3
GO:BP	**Interleukin-15-mediated signaling pathway**	GO:0035723	0.004746585	2.323618731	3	CD4,IL2RB,IL2RG
GO:BP	**Regulation of interleukin-12 production**	GO:0032655	0.008353316	2.078141096	7	ACP5,HMGB1,IL16,LAPTM5,LTB,NFKB1,TIGIT
GO:BP	**Regulation of interleukin-1 beta production**	GO:0032651	0.010527956	1.977655943	9	ACP5,CCL3,CCR5,EGR1,GBP5,HMGB1,IGF1,LPL,TNFAIP3
GO:BP	**Interleukin-10 production**	GO:0032613	0.01241493	1.906055714	7	CD28,CD34,EPX,HGF,HMGB1,SASH3,TIGIT
GO:BP	**Interleukin-4 production**	GO:0032633	0.014141994	1.84948936	5	CD28,CD3E,EPX,PRKCQ,SASH3
GO:BP	**Response to interleukin-8**	GO:0098758	0.018520782	1.732340674	2	CXCR2,EGR1

Source	Term Name	Term ID	FDR	-Log10 FDR	Intersection size	Intersections
GO:BP	**Regulation of cell adhesion**	GO:0030155	4.51E-17	16.34600683	69	ACP5,AKNA,ALOX5,ANK3,C1QTNF1,CARD11,CCL5,CCR5,CD244,CD27,CD28,CD3E,CD4,CD5,CORO1A,CTLA4,CTSG,CXCR3,DLG1,DSCAM,EPHB2,ETS1,FERMT1,FLOT1,FN1,GIMAP5,HMGB1,ICOS,IGF1,IL27RA,IL2RG,ITGA6,ITGAL,JAK3,JUP,LAG3,LAMA4,LAPTM5,LAX1,LCK,LPXN,MMP12,NDNF,NFAT5,PDE4D,PLEKHA2,PREX1,PRKCQ,PRKD2,PRLR,PTPN6,PTPRA,RAC2,RND1,RPS3,S100A10,S1PR1,SASH3,SPECC1L,SPINT2,SWAP70,TESPA1,TGM2,TIGIT,TNFAIP3,TNFAIP8L2,TNR,UBASH3B,WNT4
GO:BP	**Cell-cell adhesion**	GO:0098609	4.74E-14	13.32437688	69	AJUBA,AKNA,ALOX5,ANK3,C1QTNF1,CARD11,CCL5,CCR5,CD2,CD200,CD244,CD27,CD28,CD34,CD3E,CD4,CD5,CD93,CLDN12,CORO1A,CTLA4,CTSG,CYFIP2,DLG1,DSCAM,ETS1,FLOT1,FN1,GIMAP5,HMGB1,ICOS,IGF1,IL27RA,IL2RG,ITGA6,ITGAL,ITGB7,JAK3,JUP,LAG3,LAPTM5,LAX1,LCK,MEGF11,NFAT5,NLGN3,PDE4D,PKP3,PRKCQ,PTPN6,PTPRD,PTPRT,RAC2,RPS3,SASH3,SLC7A11,SLIT2,SPECC1L,SPINT2,SWAP70,TENM4,TESPA1,TIGIT,TNFAIP3,TNFAIP8L2,TNR,TSPAN32,UBASH3B,WNT4
KEGG	**Cell adhesion molecules**	KEGG:04514	0.000104321	3.981629971	20	CD2,CD28,CD34,CD4,CNTN1,CNTNAP1,CTLA4,H2-K1,H2-Q6,H2-Q7,ICOS,ITGA6,ITGAL,ITGB7,NLGN3,NRXN2,NRXN3,PDCD1,SELPLG,TIGIT
KEGG	**Chemokine signaling pathway**	KEGG:04062	0.011121011	1.953855747	17	ADCY7,ARRB1,CCL3,CCL4,CCL5,CCR5,CXCR2,CXCR3,CXCR5,GNAQ,GRK4,IKBKG,JAK3,NFKB1,PREX1,RAC2,RASGRP2
GO:BP	**Chemokine-mediated signaling pathway**	GO:0070098	0.013364624	1.874043246	8	CCL3,CCL4,CCL5,CCR5,CXCR2,CXCR3,CXCR5,SLIT2
GO:BP	**Cellular response to chemokine**	GO:1990869	0.023797925	1.62346091	8	CCL3,CCL4,CCL5,CCR5,CXCR2,CXCR3,CXCR5,SLIT2

Figure S6 (related to Figure 8). Loss of TCF-1 alters gene expression of post-transplanted alloactivated donor CD4 T cells: Gene ontology enrichment analysis of post-transplanted alloactivated CD4 T cell samples was conducted using the g: Profiler toolset; g:GOSt tool. Tables showing the cell death and apoptotic process-related pathways and the involved genes that were altered due to loss of TCF-1, the T cell-mediated process-related pathways and the involved genes that were altered due to loss of TCF-1 and the cytokine response and production-related pathways and the involved genes that were altered due to loss of TCF-1 are provided.
